# Interactive and passive mixed reality distraction: effects on cold pressor pain in adults

**DOI:** 10.3389/fpain.2024.1331700

**Published:** 2024-07-12

**Authors:** Jamie G. Murray, Line Caes

**Affiliations:** ^1^ School of Psychology & Neuroscience, University of Glasgow, Glasgow, United Kingdom; ^2^ Psychology, Faculty of Natural Sciences, University of Stirling, Stirling, United Kingdom

**Keywords:** pain threshold, attention focus shift, experimental design, digital space, hurt

## Abstract

While interactive distractors are predicted to be more effective in reducing acute pain than passive distractors, the underlying mechanisms remain poorly understood. Previous work using Virtual-Reality (VR) has suggested that interactive distraction may be enhanced by increasing the person's sense of immersion. Despite the possible utility of immersive VR in reducing pain, some people report being disoriented and motion sick, and it doesn't allow for interactions with environment (e.g., following instructions from medical staff). Here, we explore the role of the immersion in the effectiveness of interactive distraction by employing an alternative technology, a Mixed-Reality (MR) headset that limits disorientation by projecting virtual objects into the real world. Healthy volunteers (18–35 years) participated in two experiments employing either a between (*N* = 84) or a within-subject (*N* = 42) design to compare Interactive and Passive distraction tasks presented via MR or a standard computer display. For both experiments, a cold-pressor task was used to elicit pain, with pain tolerance and pain perception being recorded. Analysis revealed that whilst interactive distraction was more effective in reducing pain perception and increasing pain tolerance than passive distraction, the interpretation of results was sensitive to experimental design. Comparison of devices did not reveal significant differences in pain tolerance or pain intensity, while pain unpleasantness was significantly reduced during the MR task using a within-subject design. Our findings add to existing VR studies reporting little additional analgesic benefit of new, immersive technologies compared to traditional computers, but underscores the important impact the choice of experimental design can have on the interpretation of results.

## 1 Introduction

Experience of acute pain (i.e., sudden, short-lasting pain, i.e., lasting anywhere from a few minutes to less than 6 months) can have adverse psychological consequences. A common cause of acute pain—especially in childhood—are invasive medical procedures. Ineffectively managed pain during medical procedures can influence reactions to and even avoidance of future procedures (1, 2). Hence, appropriate measures need to be put in place to effectively manage acute pain (3). One such measure is distraction—a widely accepted, evidence-based pain management strategy for mitigating acute pain (4, 5). However, questions remain about the most effective form of distraction.  

Interactive distracters (i.e., multisensory activities engaging cognitive, emotional, and motor responses such as computer games) are predicted to be more effective compared to Passive distracters (i.e., those that do not engage such faculties, such as simply watching a film) because Interactive distracters require greater attentional processing (6). To date, few studies have systematically explored the efficacy of diverse types of distracters in comparison to each other, with even less work focusing on the specific mechanisms that may explain any observed differences between distractors. Moreover, the limited available evidence is mixed and inconclusive (6–9). Consequently, the choice between Interactive vs. Passive distraction remains an issue of debate (10, 11) and recent reviews have concluded that instead of continuing to compare a mix of distracters, there is an urgency to systematically dismantle the effective components of distraction techniques (4, 5, 8).  

One potential component of interest is the role of immersion which generally refers to the subjective experience of being deeply engaged and involved in an artificial environment, with a diminished awareness of the real-world surroundings (12, 13). Immersion is influenced by factors such as sensory fidelity, interactivity, and the level of engagement experienced by the user, rather than solely by the size or proximity of the display screen (14). The most immersive distraction-related tasks are delivered via Virtual Reality (VR) or Mixed Reality (MR) devices. VR involves the user being presented with a vivid virtual environment by using a head-mounted display (HMD) to shut off the physical world. By contrast, MR devices overlay the virtual environment or objects over the physical world by means of a HMD, thereby creating a “mixed” virtual/real world experience (15).  The use of VR as a possible effective non-pharmacological analgesic was demonstrated in early case studies—most notably in the development of “SnowWorld” (16). While a recent meta-analysis of existing literature suggested that the use of VR was generally associated with better pain management among patients with chronic pain (17), preliminary studies comparing VR to traditional 2D VR has revealed little additional benefit when interacting with games (18, 19), and to date, no such comparison with MR have taken place. Moreover, to our knowledge, no pain distraction research has implemented MR technology, despite its potential to overcome some limitations with VR by providing a less disorientating immersive experience and allowing for interactions with the environment [e.g., receiving instructions from medical staff or family support (20)].

To shed more light on the underlying mechanisms of Interactive distraction, in particular the role of immersion, we aimed to compare the effectiveness of Interactive vs. Passive distraction delivered by the Microsoft HoloLens MR device vs. a standard computer. We hypothesised that:   

Hypothesis 1: Pain tolerance will be higher in Interactive distraction conditions compared to Passive distraction conditions, and this task difference will be greater when delivered through MR compared to a standard computer. 
Hypothesis 2: Pain intensity and pleasantness scores will be lower in the Interactive conditions compared to Passive distraction conditions, and this difference will be greater for the task delivered through MR compared a standard computer. 

To test these hypotheses, two experimental studies were conducted, using the Cold Pressor Task (CPT) as the pain inducing stimuli. Experiment 1 employed a between-group design to explore pain perception and pain tolerance between interactive, passive, and Baseline (i.e., no distraction) conditions. Experiment 2 builds on the overall pattern of results by reducing the number of conditions (no distraction) to facilitate a direct comparison of interactive and passive conditions within subjects.

## 2 Experiment 1

### 2.1 Method

#### 2.1.1 Participants

A total of 84 undergraduate students from the University of Stirling gave informed consent (approved by the University of Stirling Psychology Ethics Committee) and were awarded course tokens in return for their participation. The mean age of participants in the Computer group was 21.97 years (SD = 2.15; range: 20–35 years) of which 21 (50%) identified as female. The mean age of the Hololens group was 20.95 (SD = 2.73; range: 18–28 years). It is important to note that we did not gather demographic data beyond sex differences including gender or race. An a-priori power analysis carried out in G* Power version 3.1.9.4 (21) for sample size estimation. An estimate of the observed effect size from similar studies (6, 19) suggested a moderate-sized effect, which informed our sample size determination. With a significance criterion of a = .05 and power of 80%, the minimum sample size needed for each group to determine a moderate sized effect was 28 for both groups. The obtained sample size of 42 participants per group is therefore more than adequate to test the study hypotheses using a mixed ANOVA. All participants reported no history of chronic pain, were not suffering from a broken hand or arm, or from contraindicated problems with circulation, blood disorders or who had a history of heart problems or frost bite. Participants with hearing or vision impairments were instructed not to enrol in the study as these factors would interfere with the use of the MR equipment, unless these impairments were corrected for by glasses or hearing aids. Participants also had to be able to read and write in English to answer written questions and understand all study instructions. Participants were randomly assigned to either the Computer condition (42 participants) or the Mixed Reality condition (42 participants). Two participants in the Mixed Reality condition did not complete all three experimental tasks and were removed from the analysis.

#### 2.1.2 Experimental procedure

**Figure 1:**
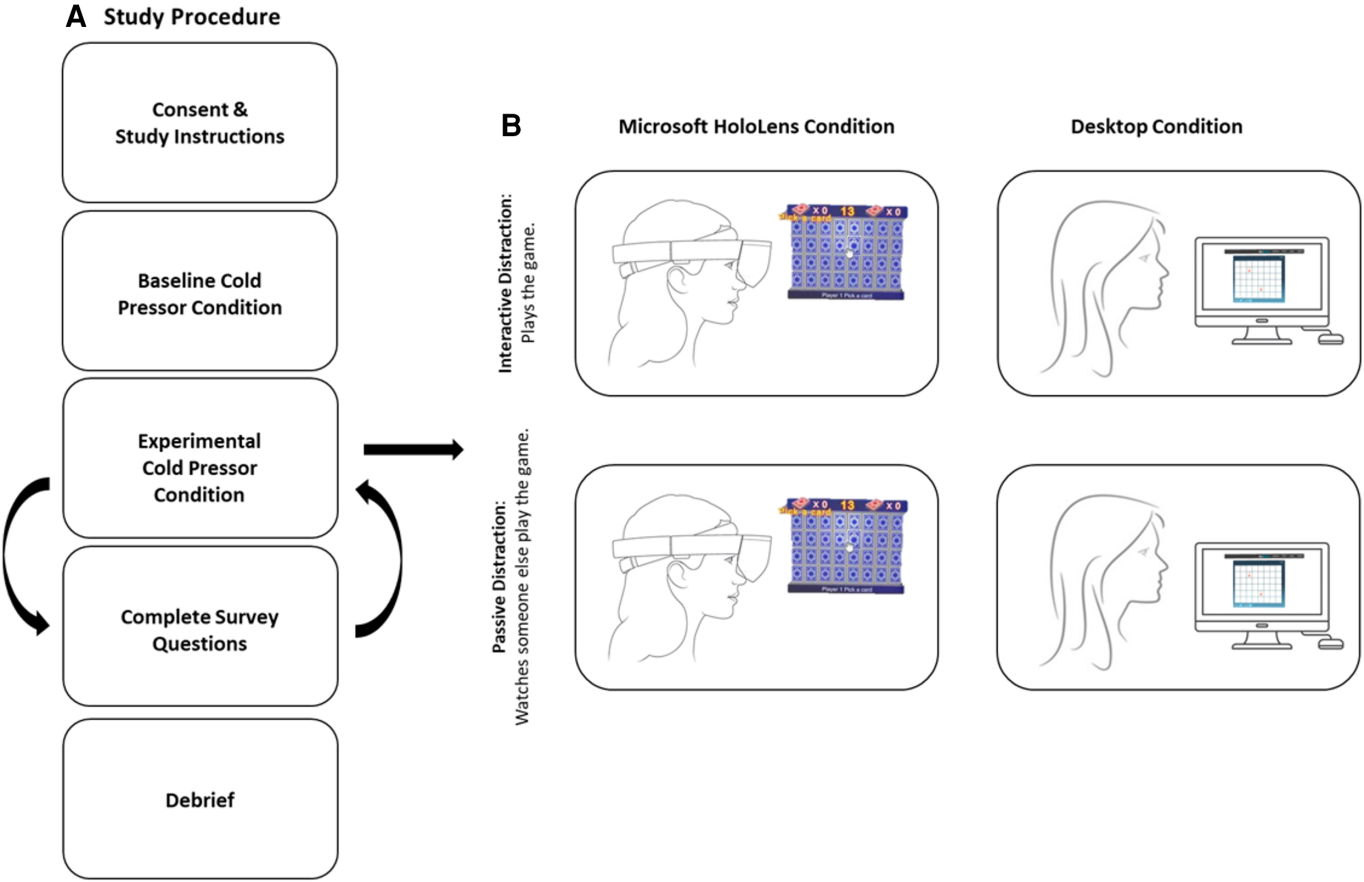
Visual representation of (**A**) overall experimental procedure and (**B**) the passive and interactive conditions across both computer and Hololens devices.

Upon arrival at the laboratory, the experimenter explained the procedure and aim of the study. After written consent was obtained, participants taking part in the Mixed Reality condition were required to complete a training session to familiarise them with the gesture-based interface. This training procedure involved playing a single round of “*BattleBrain”* to completion (the time taken to complete this round varied between participants)*.* After familiarising participants with the procedure and technology, experimenters recorded the temperature of the participants' hands using an ATP multi-function Meter (Dt-22 light) thermometer. Participants were also informed of the possibility of feeling unwell while using the Hololens and were encouraged to stop the study at any point if they experienced discomfort or any adverse effects. Participants were then asked to sit beside the cold pressor apparatus and to remove any jewellery or watches on their arms, wrists, or hands. All participants in both conditions (i.e., Computer vs. Mixed Reality Condition) took part in three cold pressor tasks: Baseline, Passive, and Interactive Distraction (for a visual representation of the study procedure see Figure 1).

Baseline: All participants (in both the Mixed Reality and Computer conditions) performed the Baseline task before the Passive and Interactive tasks. Here, participants were given the standard cold pressor instructions (as detailed above) and were given no further task instructions.

Passive Distraction: Participants were either seated in front of a laptop computer (Computer condition) or were fitted with the Mixed Reality headset (Mixed Reality condition). Participants were then instructed to watch a pre-recorded video of the experimenter playing PAIRS against a computer AI directly before submerging their hand in water. The trial began after the experimenter entered the adjacent room and knocked on the window to indicate that the participant was to submerge their hand in water. The full length of the videos (in both Mixed Reality and Computer conditions) was 5 min, which was longer than the maximum length (4 min) that participants were asked to keep their hand submerged in water. All other procedures were identical to the baseline.

Interactive Distraction: Participants were seated in front of the laptop computer (Computer condition) or were fitted with the Mixed Reality headset (Mixed Reality condition). Participants were then asked to begin playing the game. Once participants began playing the game (either on the computer or in Mixed Reality), the experimenter entered the adjacent room and signalled for the trial to begin, at which point the participant's non-dominant hand was submerged in water. All other procedures were identical to the baseline.

Task order was counterbalanced across participants, so that half of the participants in each condition took part in the Passive Distraction task first after Baseline and the other half completed the Interactive Distraction task first after the Baseline. The counterbalancing of submerged hand (left/right) and task (Baseline, Passive, Interactive), however, was dependent on participants dominant hand which had to be free to control the game during the Interactive condition. To be clear, if the Interactive task followed the Baseline task, participants were required to submerge their dominant hand in water during Baseline. If the Interactive task followed the Passive task, participants submerged their non-dominant hand in water during the Baseline and dominant hand during the Passive task.

The cold pressor procedure was identical for all three experimental tasks (Baseline, Passive, and Interactive). Once participants were positioned beside the cold pressor, the experimenters explained that upon hearing a knock, they were to lower their hand in the water up until their wrist bent. Participants were to hold their unclenched hand in position until the experimenter indicated (*via* a second knock) it was time to remove their hand. However, participants were reminded that they could take their hand out of the water at any time if they felt that their hand was too uncomfortable or too painful. Participants were then asked to repeat the instructions to confirm that they understood the task. Next, the experimenter left the room and entered an adjacent room with a one-way observation mirror. The experimenter then knocked on the window to signal that the participant was to lower their hand. The duration of hand submersion was recorded via a stopwatch which began recording from the initial submersion to the full removal of the participant's hand. A maximal duration of 4 min was set for each experimental task (i.e., the recommended ceiling for paediatric cold pressor studies (22). Once the maximal time limit was reached, the experimenter knocked a second time on the window to signal that the participant was to remove their hand from the cooled water. In case the participant had taken their hand out before the 4 min limit was reached, the experimenter entered the room right after noticing the participant took their hand out.

After each cold pressor task, participants were asked to dry their hands and leave the testing room. Participants were then asked to complete three questionnaires measuring pain intensity and pain unpleasantness. Participants were instructed to complete the questionnaires promptly in order to minimize memory decay and ensure that accurate recall of experiences during the experimental procedure. There was no set time between the conditions, but before taking part in the next experimental condition, the temperature of each participant's submerged hand was taken to ensure that the temperature had returned to baseline levels recorded at the beginning of the study. After all three tasks and questionnaires were completed, participants were fully debriefed about the aims and objectives of the study.

#### 2.1.3 Materials and measures

##### 2.1.3.1 Cold pressor task

A Cold Pressor Task (CPT) with water temperature set to 4 (±.01)° was used to induce pain in adult participants. The cold pressor task was chosen as the pain-inducing task as it is a well-established, ethically approved task that has been shown to be suitable for eliciting acute pain experience in adults (23). The cold pressor device consisted of a commercially manufactured electronic cooler measuring 30 cm wide, 40 cm long, and 45 cm high. Participants were instructed to lower their hand into the water through a rectangular opening in the lid (16 cm by 10 cm with a depth of 16 cm). Participants were asked to submerge their full hand into the water, until the water reached just above the wrist. To minimise fluctuation on their hand temperature, participants were asked to keep their hand in an unclenched position (i.e., spread the fingers open and not form a fist) and not move their hand. In addition, the water in the tank was continuously circulated by a pump to prevent local warming. Participants kept their hand submerged for up to 4 min (or until they began to feel that the pain was unbearable).

##### 2.1.3.2 Microsoft hololens

The mixed reality headset was a 1st generation Microsoft Hololens. The Hololens is a pair of mixed reality smart-glasses that can project high quality 3D virtual objects into real world environments and can be controlled via a series of gesture, gaze and voice commands. Resolution of the device is set to 1280 × 720 pixels per eye which is equal to High Definition (HD) resolution screens. The Microsoft Hololens has a refresh rate of 60 hz with a 30° (horizontal) by 17.5° (vertical) field of view. On connection, the Hololens could display either videogame performance (Interactive distraction task) and the pre-recorded videogame footage (Passive distraction task). No auditory effects of the game were presented as volume on the device was muted. In comparison, a standard desktop PC was used for the Computer Condition and controlled using a mouse. Again, volume was muted for both the Passive and Interactive tasks.

##### 2.1.3.3 Distraction task

The card game PAIRS was used in both the Mixed Reality and Computer conditions. This card game was chosen because a similar version is available to play on the Hololens and computer and represents a task that is cognitively demanding because it actively involves a person's working memory. For the Microsoft Hololens, the specific PAIRS game used was called “BattleBrain” (published by Jopacus Parrott) comprising of a 6 × 8 grid of coloured shapes. Here, participants were tasked with rotating cards and remembering pairs of shapes whilst competing against a computer AI. In the computer condition, a similar PAIRS game (published by dkmGames: www.dkmgames.com/Memory/Pairs.php) was used comprising of a 6 × 8 grid of coloured shapes. Again, participants were competing against a computer AI. For the Passive task participants either viewed a pre-recorded video of the experimenter playing the game against the AI using the Microsoft Hololens or Computer, whilst the participant's hand was submerged in cold water.

##### 2.1.3.4 Self-report

After each CPT (Baseline, Passive, Interactive), participants were requested to provide a written report on pain intensity, and pain pleasantness. For pain intensity, participants were asked how much pain they had experienced during the CPT. Participants rated their pain by means of a 11point Numerical Rating Scale (NRS) from 0 (“no pain”) to 10 (“a lot of pain”) (24). For unpleasantness, participants were asked how “unpleasant/horrible/yucky” the pain was when their hand was in the water. Again, participants rated their unpleasantness on an 11-point rating scale from 0 (“not all unpleasant/horrible/yucky”) to 10 (“most unpleasant/horrible/yucky”).

#### 2.1.4 Data analysis

Statistical analyses were performed with Jamovi (25). Descriptive statistics and Mixed ANOVAs were performed to test both hypotheses 2-tailed. Significant main effects or interactions observed from the ANOVA were followed with *post-hoc* paired samples *t*-tests with *p* values adjusted for multiple comparisons using the Bonferroni correction.

### 2.2 Results

#### 2.2.1 Pain tolerance

**Figure 2:**
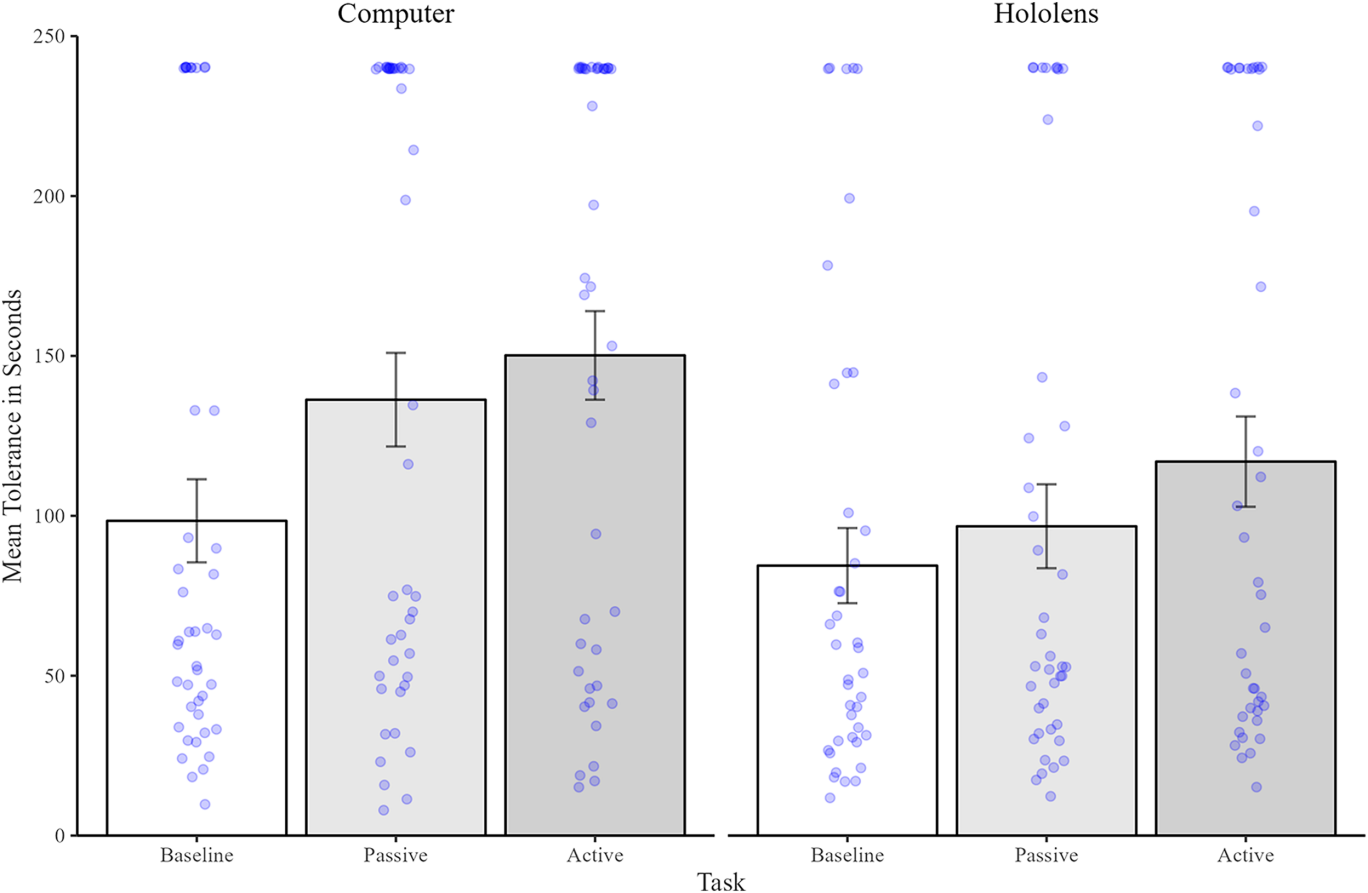
Mean (and standard errors) of pain tolerance time (in seconds) for the baseline condition (shown in white), passive condition (shown in dark grey), and interactive condition (shown in light grey). Individual data points are shown as blue dots.

Figure 2 represents the data from the mean tolerance times for each task and device. To investigate differences in mean tolerance time across tasks and devices, the data was submitted to a mixed 2 × 3 Mixed ANOVA with one between group factor of Device [Computer/Mixed Reality], and one within subject factor of Task [Baseline/Passive/Interactive]. Results revealed a significant main effect of Task [*F*(2, 80) = 19.5, *p* < .001, *η*2 = .20] but no significant main effect of Device [*F*(2,80) = 2.78, *p* = ns] nor a Task by Device interaction [*F*(2, 80) = 0.15, *p* = ns]. Pairwise *t*-tests (with Bonferroni adjusted *p*-values) revealed that mean tolerance durations were significantly shorter at Baseline (mean = 91.6 s, sd = 79.37) compared to both the Passive (mean = 117 s, SD = 90.91) task; [*t*(81) = 3.70, *p* < .001; *d* = .30] and the Interactive task (mean = 133.96 s, SD = 90.56) task [*t*(81) = 5.76, *p* < .001; *d* = .50]. Critically, durations were significantly longer in the Interactive compared to Passive task [*t*(81) = 2.34, *p* = .04; *d* = .19].

#### 2.2.2 Pain intensity

**Figure 3:**
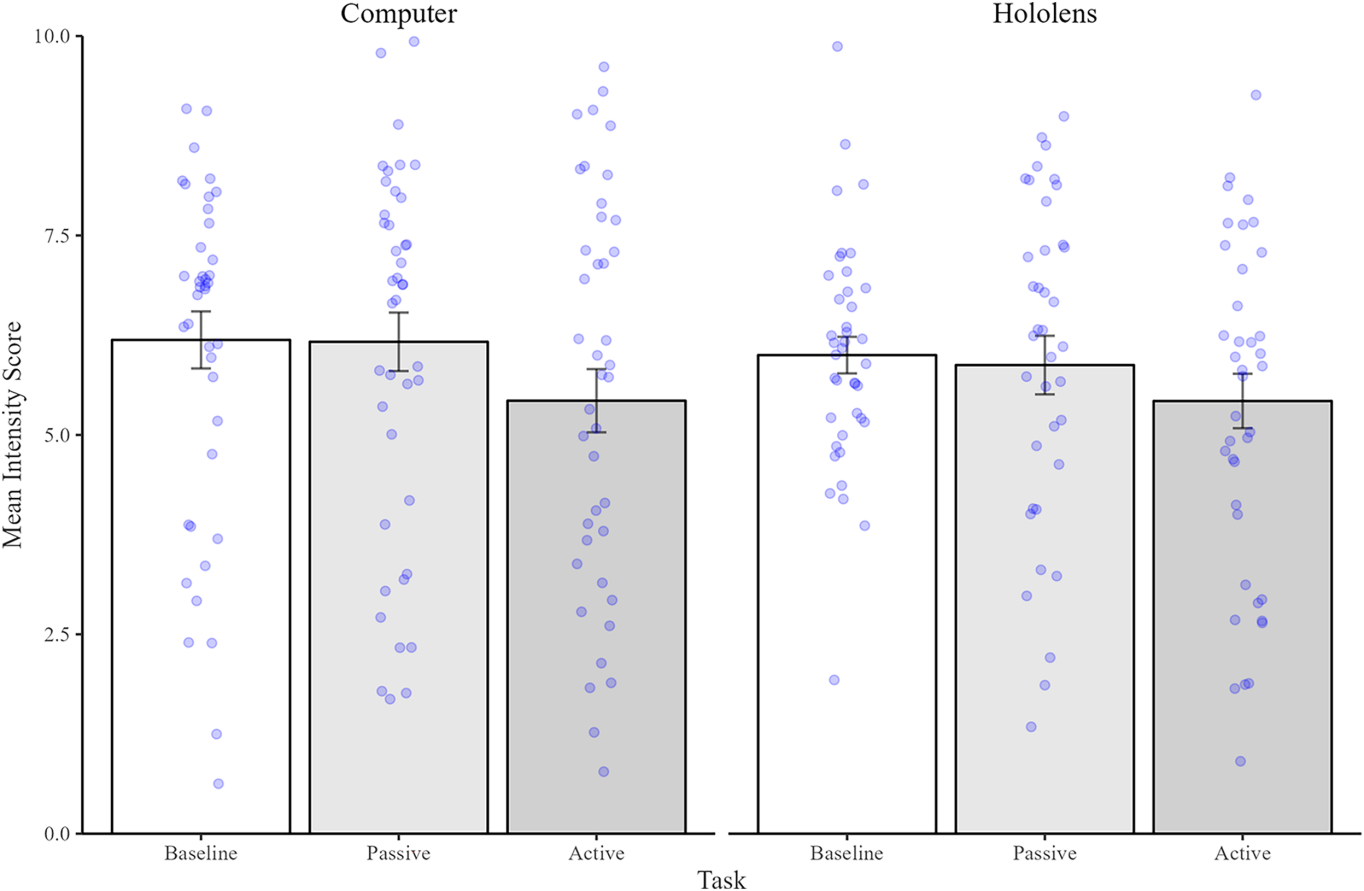
Mean (and standard errors) of intensity scores with 0 being the least intense and 10 being the most intense. Bars represent the Baseline condition (shown in white), Passive Condition (shown in dark grey), and Interactive condition (shown in light grey). Individual data points are shown as blue dots.

Figure 3 illustrates mean intensity scores reported by participants. A 2 × 3 Mixed ANOVA (Device by Task) revealed a significant main effect of Task [*F*(2, 80) = 5.37, *p* = .007, *η*2 = .06] but no main effect of Device [*F*(2,80) = .15, *p* = ns], nor any significant interaction [*F*(2,80) = .21, *p* = ns]. *Post hoc* analyses of the main effect of Task confirmed that participants rated their pain as being as equally intense in both the Baseline (mean = 6.10, SD = 1.94) and Passive tasks (mean = 6.02, SD = 2.34) tasks [*t*(81) = .33, *p* = ns]. However, Pain was felt as being significantly less intense in the Interactive task (mean = 4.43, SD = 2.36) compared to both the Baseline [*t*(81) = 2.99, *p* = .01, *d* = .31] and Passive tasks [*t*(81) = 2.66, *p* = .03, *d* = .25].

#### 2.2.3 Unpleasantness

**Figure 4:**
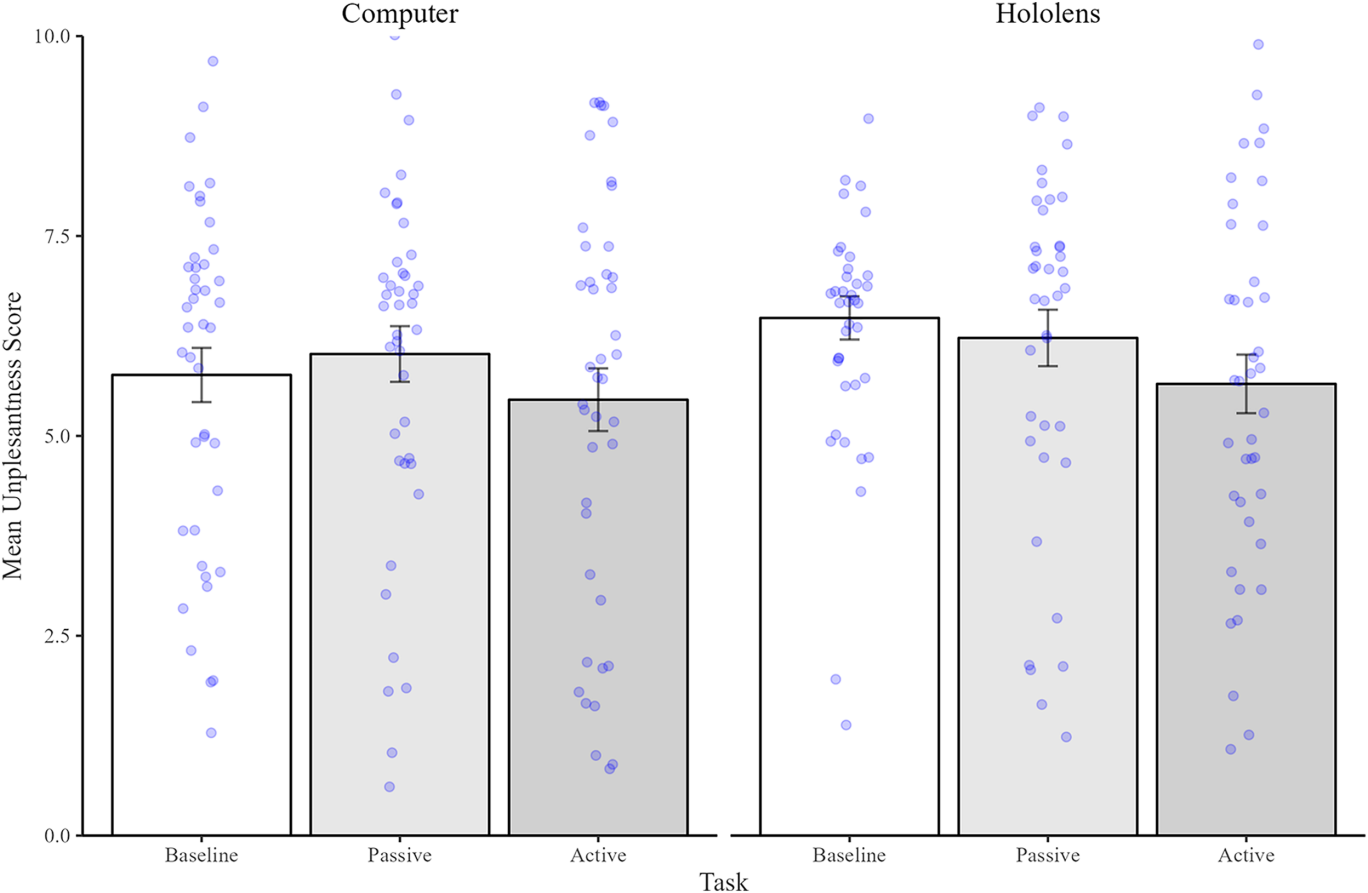
Mean (and standard errors) unpleasantness scores with 0 being least unpleasant and 10 being the most unpleasant. Bars represent the Baseline condition (shown in white), Passive condition (shown in dark grey), and Interactive Condition (shown in light grey). Individual data points are shown as blue dots.

Figure 4 illustrates the mean unpleasantness scores between experimental Tasks and Devices. Here, the Mixed ANOVA analysis of mean unpleasantness scores revealed a significant main effect of Task [*F*(2, 80) = 4.84, *p* = .009, *η*2 = .06] but no significant main effect of Device [*F*(1,80) = .75, *p* = ns] nor any significant interaction [*F*(2,80) = .98, *p* = ns], suggesting that pain unpleasantness varied between tasks but not between devices. *Post hoc* analyses of the main effect of Task confirmed that participants rated their pain as being equally unpleasant in both the Baseline (mean = 6.11, SD = 1.99) and Passive (mean = 6.12, SD = 2.24) tasks [*t*(81) = .03, *p* = ns]. However, pain was rated as significantly less unpleasant in the Interactive task (mean = 5.55, SD = 2.42) compared to both the Baseline [*t*(81) = 2.45, *p* = .05, *d *= .25] and Passive [*t*(81) = 2.80, *p* = .02, *d* = .24] tasks.

### 2.3 Experiment 1 discussion

The results from Experiment 1 are clear: Interactive distraction was the most effective task for increasing pain tolerance, reducing unpleasantness of the painful event, and resulting in less painful intensity compared to both Passive distraction and no distraction. Interestingly, the results also suggest that whilst Passive distraction increases pain tolerance time, it had little effect on the perception of the painful event (i.e., pleasantness and intensity) compared to undergoing the painful event without a distraction. Critically, neither pain tolerance nor perception statistically differed between MR and the Computer condition, mirroring previous pain distraction studies employing Virtual Reality devices (18, 19, 26).

The use of a between-group design allowed us to effectively compare both Interactive and Passive distraction to a Baseline—no distraction condition, whilst limiting the number of painful events experienced by participants. The between-group design also allowed us to compare one group of participants performing the task on the MR device to another group using the Computer, although individual differences in either pain perception or previous MR experiences could have confounded the findings. For a comprehensive understanding of the role of immersion, a direct within participant comparison of devices is critical. For instance, it is possible that individuals may respond to, or perceive pain differently, depending on whether they had previously used MR device or the Computer for distraction. In addition, the tolerance data also appears to show a non-significant but numerically shorter tolerance time when using the MR device across all three conditions. This pattern of data could be partially explained by diminished statistical power because of our between-group design. Given these reservations and to provide a more a more direct comparison of MR and computer devices, we therefore repeated our experiment using a within subject design. To limit the number of repeated painful events experienced by participants in a single experiment, we excluded the Baseline condition from the second, within-subject experiment.

## 3 Experiment 2

### 3.1 Method

#### 3.1.1 Participants

Forty-two undergraduate students from the University of Stirling gave informed consent (approved by the University of Stirling Psychology Ethics Committee) and were awarded course tokens in return for their participation. The mean age of the participants was 19.95 years (SD = 2.16, range: 18–28) of which 31 (74%) identified as female. Again, no additional demographic information beyond sex was collected. An a-priori power analysis using G* Power version 3.1.9.4 (21) was carried out to estimate the sample size required for the study employing a repeated measures ANOVA. Again, using a moderate sized effect, an *a* = .05, and power of 80%, it was determined that a minimum sample of 34 participants was required to determine a moderate sized effect. Therefore, our sample of 42 participants is more than sufficient to test our study hypotheses using a repeated measures ANOVA. All participants reported no history of chronic pain, were not suffering from a broken hand or arm, or from contraindicated problems with circulation, blood disorders or who had a history of heart problems or frost bite. Participants with hearing or vision impairments were instructed not to enrol in the study as these factors would interfere with the use of the MR equipment, unless these impairments were corrected for by glasses or hearing aids. Participants also had to be able to read and write in English to answer written questions and understand all study instructions.

#### 3.1.2 Experimental procedure

The experimental procedure for Experiment 2 was almost identical to Experiment 1 except for a few key changes. First, we excluded the Baseline task so that every participant took part in both the Interactive and Passive tasks on the Computer and the Hololens. Second, both tasks (Interactive/Passive) and devices (Computer/Hololens) were counterbalanced across participants. Again, the counterbalancing of submerged hand (left/right) and task (Passive, Interactive), however, was dependent on participants dominant hand which had to be free to control the game during the Interactive condition. To be clear, if the Interactive task followed the Passive task, participants submerged their non-dominant hand in water during the Passive task.

#### 3.1.3 Materials and measures

Materials were identical to those used in Experiment 1 but with the exclusion of the Baseline task.

#### 3.1.4 Data analysis

Statistical analyses were performed with Jamovi (25). Descriptive statistics and Mixed ANOVAs were performed to test the hypotheses 2-tailed. Significant main effects or interactions observed from the ANOVA were followed with *post-hoc* paired samples *t*-tests with *p* values adjusted for multiple comparisons using the Bonferroni correction.

### 3.2 Results

#### 3.2.1 Pain tolerance

**Figure 5:**
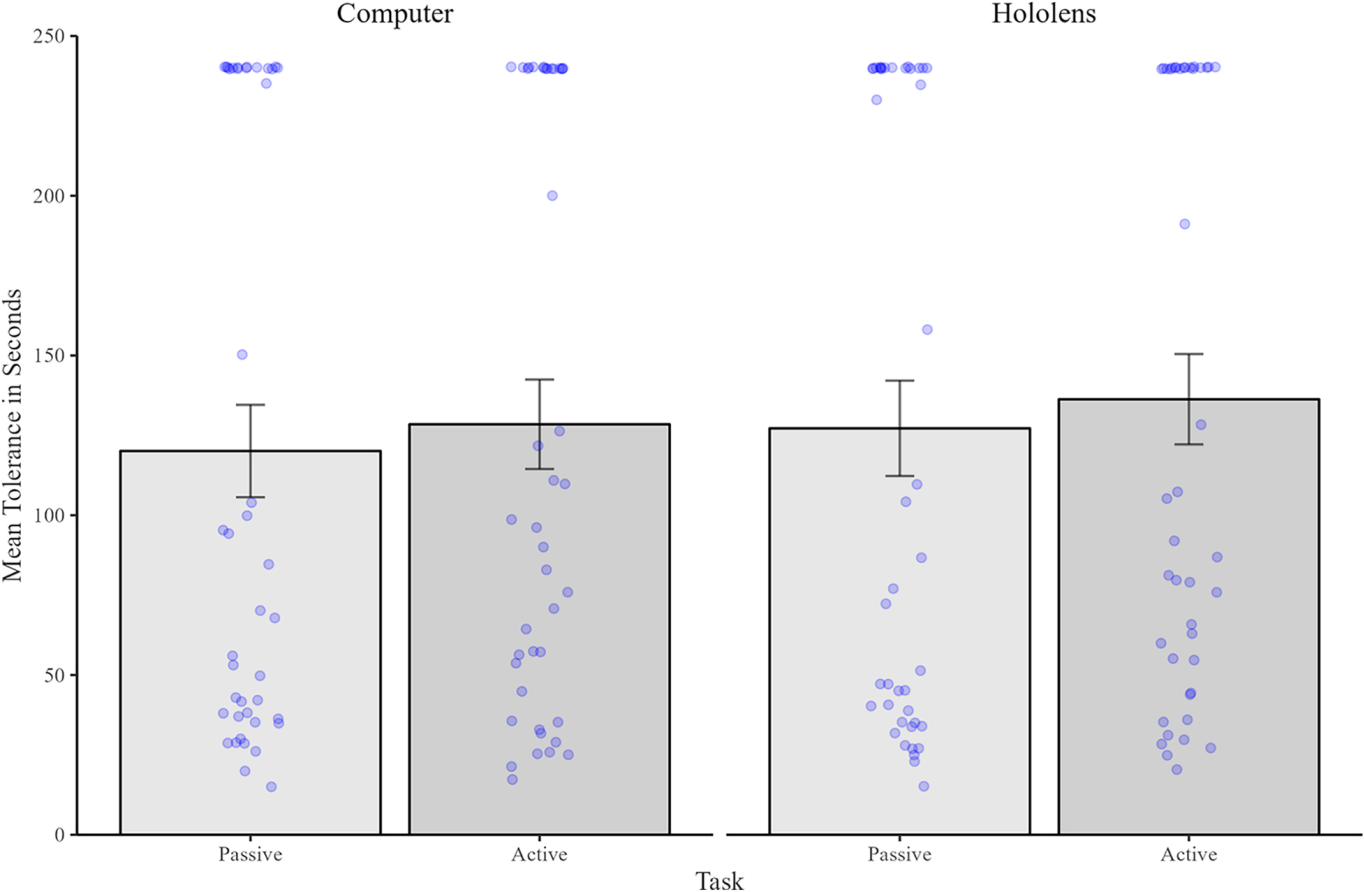
Mean (and standard errors) pain tolerance time (in seconds) for the passive condition (shown in dark grey), and interactive condition (shown in light grey). Individual data points are shown as blue dots.

Pain tolerance means (in seconds) can be seen in Figure 5 which reveals very little difference in tolerance time between tasks or device. A repeated measures ANOVA revealed no significant main effects of Task [*F*(1,41) = 1.65, *p* = ns], Device [*F*(1,41) = 1.72, *p* = ns], nor any significant interaction [*F*(1,41) = .02, *p* = ns].

#### 3.2.2 Pain intensity

**Figure 6:**
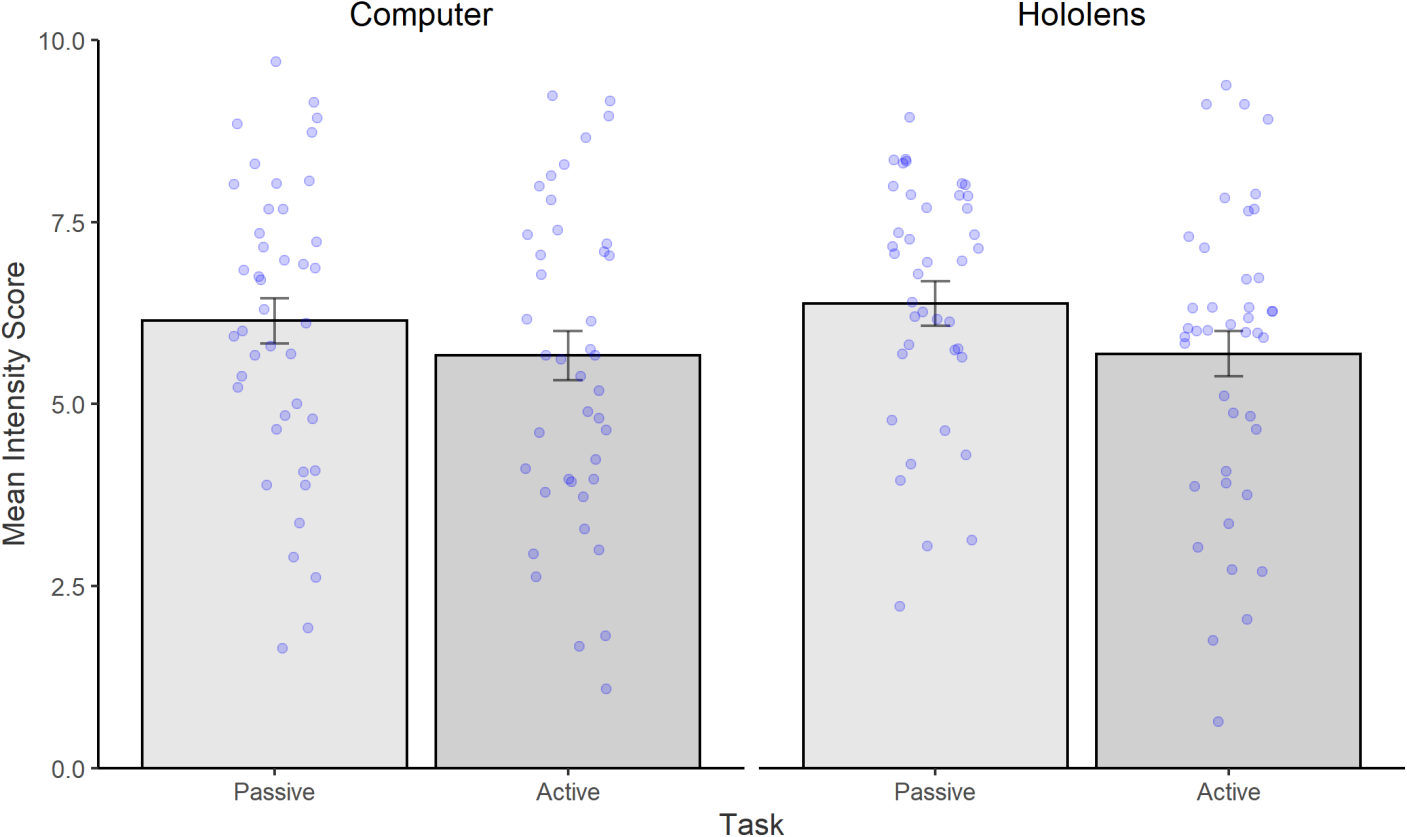
Mean (and standard errors) of intensity scores with 0 being the least intense and 10 being the most intense. Bars represent the Passive Condition (shown in dark grey), and Interactive condition (shown in light grey). Individual data points are shown as blue dots.

Figure 6 illustrates mean intensity scores. Analysis revealed a significant main effect of Task [*F*(1,41) = 4.62, *p* = .03, *η*2 = .10], indicating that pain was reported as being significantly less intense in the Interactive task (mean = 5.68, SD = 2.09) compared to the Passive task (mean = 6.26, SD = 1.98). However, intensity did not differ between Devices [*F*(1,41) = .6, *p* = ns], nor was there any significant interaction between Task and Device [*F*(1,41) = .46, *p* = ns].

#### 3.2.3 Unpleasantness

**Figure 7:**
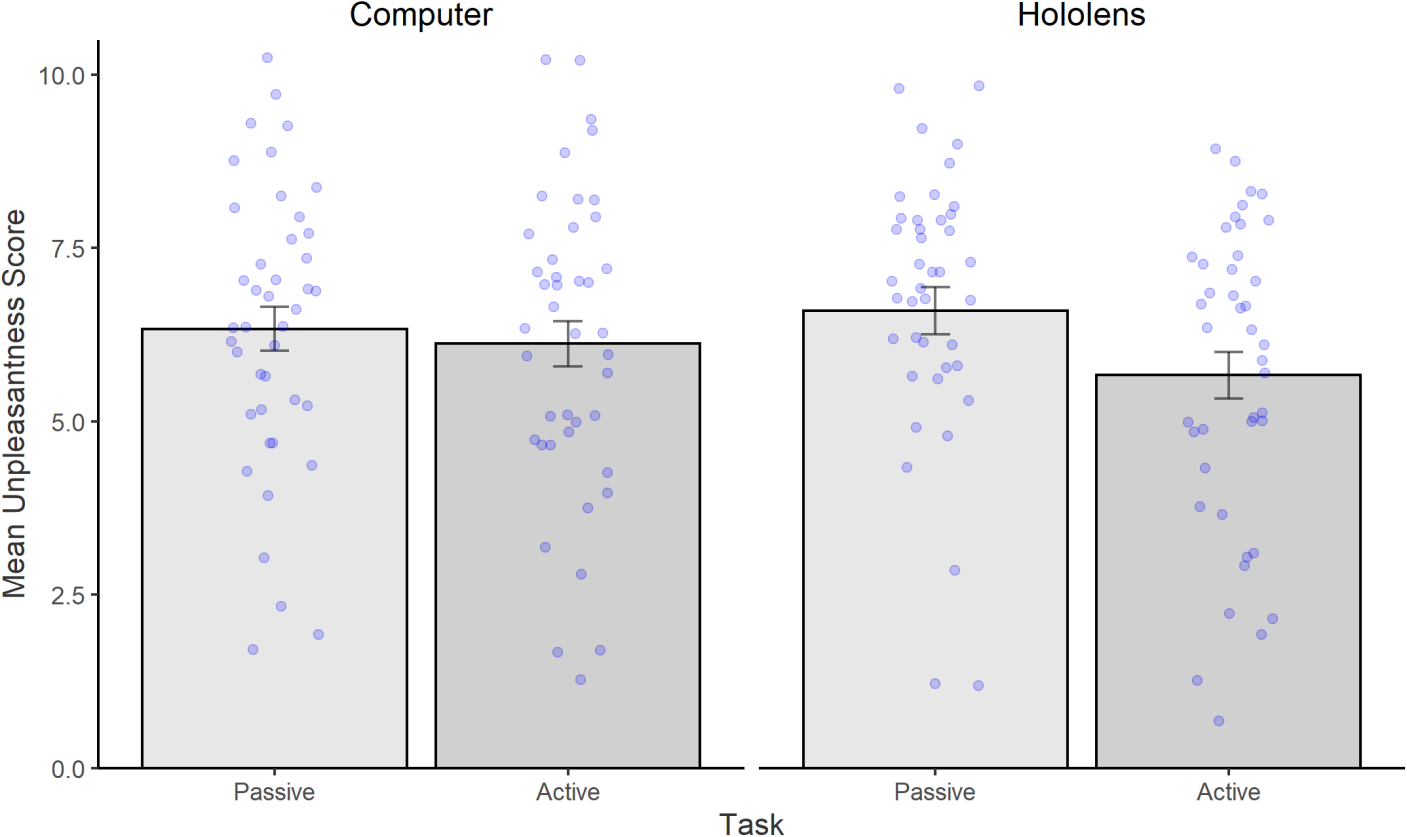
Mean (and standard errors) unpleasantness scores with 0 being least unpleasant and 10 being the most unpleasant. Bars represent the Passive condition (shown in dark grey), and Interactive Condition (shown in light grey). Individual data points are shown as blue dots.

Figure 7 shows the mean unpleasantness scores. A two-way analysis of variance yielded a marginally significant main effect of Task [*F*(1,41) = 3.94, *p* = .05, *η*2 = .08] but no main effect of Device [*F*(1,41) = .17, *p* = ns]. Critically, results also yielded a significant interaction between Task and Device [*F*(1,41) = 8.15, *p* < .01, *η*2 = .16]. *Post-hoc* tests confirmed that the interaction was driven by significantly reduced unpleasantness in the Interactive task (mean = 5.67, SD = 2.18) compared to Passive task (mean = 6.60, SD = 2.21) on the Hololens [*t*(42) = 2.68, *p* = .02, *d* = .42], whereas on the Computer there was no significant differences in pleasantness scores between the Interactive (mean = 6.12, SD = 2.13) and Passive (mean = 6.33, SD = 2.04) tasks [*t*(42) = .51, *p* = ns].

## 4 General discussion

The aim of the current study was to explore the moderating role of immersion in distraction effectiveness, thereby enhancing our understanding of the underlying mechanism of Interactive vs. Passive distraction techniques. Across two experiments, our hypotheses were only partly confirmed.

With respect to hypothesis 1, we found evidence, using the between-group design only, that pain tolerance was higher in the Interactive distraction condition compared to both Passive distraction and Baseline (Experiment 1). Critically, we failed to detect a similar increase in pain tolerance in our within subject design (Experiment 2), and in neither design was pain tolerance influenced by the device used to deliver distraction.

A similar pattern of results was observed for the pain intensity outcome of hypothesis 2, with pain intensity scores lower in the Interactive Distraction conditions regardless of the device or experimental design. Pain unpleasantness ratings, by contrast, were affected by study design and device—the within subject design resulted in reduced pain unpleasantness for Interactive distraction delivered using MR only, but no such reduction was observed in the between-subject design. The results appear to suggest that whereas pain intensity is generally lower overall for interactive distraction, they tend to rate the painful experience as less unpleasant during the interactive MR task.

While Interactive distraction appears more effective than both Passive distraction and no distraction, the pattern of results is largely dependent on the type of design (between/within subject) and, to a lesser extent, device (MR/computer) used. Our comparison of between- and within-subject designs suggests that the effectiveness of interactive distraction is substantially moderated by situational and individual differences. For instance, the significant variability observed in the data points likely reflects individual differences in pain sensitivity, such as anxiety or catastrophizing, as well as potential variation in engagement or buy-in with the distraction tasks employed. Awareness of these potentially moderating factors is important in the context of the wide variety of designs used to evaluate the impact of interactive vs. passive distraction, and hence could explain the mixed evidence for the effectiveness of interactive distraction within the literature (10, 11, 27). Consequently, our findings regarding the effect of interactive distraction on reducing the experience of pain may not be readily generalizable to more controlled situations (for example, in clinical settings) whereby situational differences are not effectively controlled.

Given that MR reduced pain unpleasantness experiences but did not result in greater pain tolerance or lesser pain intensity compared to computers, suggests that our use of MR may not have resulted in successful distraction as anticipated. Our results are, however, consistent with previous VR distraction studies that observe little additional benefit of head mounted VR distraction compared to 2D interactive distraction when managing acute pain (18, 19, 26). Given that head-mounted MR devices are relatively expensive, these findings are of relevance for clinical purposes, as our findings (taken together with previous literature) suggest that virtual environments displayed on computer monitors may provide equally effective pain management as more expensive head mounted VR and MR devices.

One important caveat in relation to immersion, however, is that our choice of game may have minimised the potential immersive advantage associated with MR. We choose our game influenced by a number of factors including availability of similar games available across both formats, general familiarity with the card game “Memory” of which PAIRs and BattleBrain are variants, evidence that working memory tasks are effective in distracting attention generally (28) and pain in particular (29). However, we acknowledge that the game used here may not have fully immersed participants compared to other MR experiences and therefore our conclusions about the role of immersion are specific to the game used in this study.

To our knowledge, this was the first study to use head mounted MR rather than VR. While we did not directly compare MR with VR, our findings are generally comparable to studies implementing VR technology, which could have important clinical considerations as VR applications are not without limitations. For example, a recent review article highlighted how VR can create experiences that are too immersive or detached from reality in some individuals, resulting in motion sickness and physical disorientation (20, 30). These side effects of a complete immersive experience introduced by VR could be of relevance for painful medical procedures, where individuals might need to be able to interact with their environment to receive support or instructions from others (e.g., medical staff or family). In such circumstance, MR could provide an equally effective alternative means of distraction with similar benefits to VR but in a less immersive and demanding environment (7), avoiding issues with disorientation, over immersion and being cut off from parents, caregivers or medical staff. Future investigation of pain distraction should therefore also incorporate the use of MR as a viable alternative to those patients likely to be sensitive to disorientation and motion sickness.

Regardless of the type of device used, by using a well-controlled and systematic comparison design, data from both experiments demonstrates that Interactive distraction is more effective for pain management than Passive distraction. Our results further validate recent models of pain and attention recognising the dynamic interplay between pain and other environmental stimuli (31). Indeed, our Interactive distraction condition involved a cognitively demanding working memory game that, theoretically, should have engaged greater attentional resources than our Passive distraction. The results are consistent with previous experimental pain distraction studies among adults examining the influence of cognitively demanding tasks (7, 29, 32–34). A major limitation of existing studies, however, concerns the differing nature of Interactive and Passive stimuli. Stimuli will often differ on various dimensions beyond whether Interactive or Passive engagement is needed, and it is difficult to determine whether the differences (or lack of differences) found between conditions is due to the attention-grabbing nature of the stimuli. The current study overcomes this limitation by ensuring the stimuli used in the Interactive and Passive distraction conditions were the same and only differed in their level of interactive engagement. Our results provide further evidence in support of Interactive distraction in reducing pain perception regardless of how the distraction is delivered to participants.

Our findings also further support the need, identified in a recent review of the VR and pain management literature for future studies of VR (and now MR), to focus on how the different dimensions of the virtual experience contribute to the potential analgesic effects of Interactive distraction—including immersion, presence, and interactivity. According to Trost and colleague's (35) heuristic model, each dimension of the user experience will contribute to the level of engagement with the virtual distraction; although exactly how each dimension contributes independently, or how they interact, to reduce different aspects of a pain experience (i.e., physiological, behavioural, social, or emotional) remains unknown. Indeed, our study design in which immersion was manipulated using different devices posed a challenge in teasing apart the independent contributions of these dimensions. Here, we opted to manipulate immersion using different devices rather than relying on subjective measures, considering the difficulties highlighted by Mutterlein and Hess (36) in subjective assessment. Regardless, we acknowledge that this decision limits our ability to draw firm conclusions about the role of immersion in pain distraction and suggest that future studies incorporate either subjective or objective measures of immersion to better understand its impact on pain perception.

This heuristic model also provides a useful framework for setting our data in context. For example, although MR and Computer Monitor distraction tasks were equally effective in reducing pain in both our experimental designs, the increased presence and immersion afforded by head mounted virtual reality may provide a stronger analgesic effect under alternative situations [i.e., as part of chronic pain management or under certain clinical procedures—see (35)] for a review). We would argue, however, that when the VR heuristic model is used as a basis to investigate distraction mechanisms, task demand should be incorporated as an additional relevant factor. Previous pain distraction studies highlight the importance of the task being performed during interactive distraction, with cognitively demanding tasks (e.g., working memory tasks) observed to be the most effective at reducing pain (8, 29, 37, 38). Our results highlight the importance of working memory type distraction tasks. However, the specific nature of the task (e.g., cognitive demands, level of immersion) may play a more crucial role than the delivery medium itself (MR, VR, or monitor). Identifying tasks that are cognitively engaging yet still promote a strong sense of immersion within virtual environments may yield maximal pain distraction benefits and warrants further investigation.

The study findings need to be considered in the light of several limitations. First, it is important to acknowledge the multifaceted nature of pain perception and modulation. Whilst distraction is indeed one coping mechanism that can influence pain perception, it is not the sole or universally effective approach for pain management (39). It is essential to acknowledge that pain modulation involves the complex interplay between physiological, psychological, and environmental factors. Distraction, while potentially effective in some cases, may not always lead to significant changes in pain threshold, as pain perception is influenced by a multitude of factors. Individual differences in pain sensitivity, cognitive appraisal of pain, and emotional state can also play a significant role in shaping pain experience (40, 41). Therefore, while our findings demonstrate the efficacy of distraction delivered via computer and mixed reality interventions in increasing pain thresholds, it is crucial to interpret these results within the broader context of pain modulation mechanisms. Future research should continue to explore the interactions between distraction and other coping strategies, as well as their combined effects on pain perception to develop a more comprehensive approach to pain management. Lastly, while subjective immersion levels could vary widely across individual, these were not assessed. Rather immersion was assumed—based on existing evidence—by using an MR device rather than a 2D computer. Future studies exploring the impact of subjective immersion levels are required for a comprehensive understanding of how different technologies impact acute pain management.

The lack of replication between Experiments 1 and 2 of the interactive distraction effect on tolerance time was also unexpected and may be attributed to differences in sample characteristics or methodological nuances between experiments. The effect sizes observed were also small to moderate suggesting that although not large, interactive pain distraction has a measurable impact on pain perception. Indeed, even small reductions in pain intensity could be meaningful for individuals undergoing painful medical procedures. Understanding that interactive pain distraction yields small to moderate effects also allow for a more tailored approach to intervention—for example, while not universally effective for everyone, certain individuals may benefit significantly from this intervention. Identification of specific patient characteristics or contextual factors that moderate the effectiveness of pain distraction techniques can help personalize treatment. While the study designs of both experiments have been carefully chosen and the findings of experiment 1 drove the focus on comparing passive vs. interactive distraction in experiment 2, the lack of baseline condition in experiment 2 needs to be considered as a limitation potentially impacting the differences in findings and lack of replication between the two experiments.

Replication of the findings is needed in a more diverse sample and across the lifespan. For instance, it would be of particular interest to explore if the findings apply to a younger population of children and adolescents, and in clinical settings (e.g., vaccinations, blood draws, burn wound dressing changes). We also did not account for individual difference in participants typical way of coping with pain (i.e., approach vs. avoidance coping), or individual preferences for distraction type (while not systematically assessed anecdotally participants expressed preference that were countering our anticipations: i.e., preferring the computer task over the Hololens or the Passive distraction over the Interactive distraction). It is plausible that individual differences in preferred pain coping strategies could moderate the effectiveness of forced usage of Interactive vs. Passive distraction. Our data is also limited by the decision to remove all sound from the game and videos used to be able to measure the direct effect of the cognitively challenging aspect of the task. Such absence of sound, however, may have affected the effectiveness of our distractors and limited the level of immersion experienced by participants (42). Further work is required to explore how different sensory elements (i.e., auditory, visual and physical elements) within MR contribute to the effectiveness of a specific distractor task. Lastly, only one game was used across both experiments, i.e., “Battle Brain”. Although this game was chosen because it was cognitively demanding, alternative MR games may provide greater levels of engagement or create a greater sense of presence (i.e., subjective sense of being involved and feeling part of a virtual environment).

The aim of the current study was to investigate the moderating role of immersion for increasing distraction effectiveness. The results of both between-group and within-experiment designs partially support the view that interactive distraction using variants of a card matching game paradigm was more effective in mediating pain perception compared to passive viewing. However, we found little additive benefit (other than decreased pain unpleasantness ratings) of the head-mounted MR presentation compared to traditional 2D computer monitors for these specific game variants. Importantly, our data suggests that the choice of experimental design should be carefully considered in future pain distraction studies aiming to unravel the underlying mechanisms of distraction effectiveness.

## Data Availability

The datasets presented in this study can be found in online repositories. The names of the repository/repositories and accession number(s) can be found below: https://osf.io/c8t53/?view_only=c0814dd2070f4794ae5a1ea3c7dd6473.
